# Failing to palpate femoral pulses in adult hypertensive patients may lead to diagnostic wandering and major cerebrovascular events in cases of undetected aortic coarctation

**DOI:** 10.1038/s41371-022-00687-9

**Published:** 2022-04-19

**Authors:** Audrey Delarue, Alexis F. Guedon, Alexandre Boutigny, Nassim Mohamedi, Benjamin Magnan, Annie Vovelle, Guy Amah, Philippe Bonnin

**Affiliations:** 1 Université de Paris Cité, AP-HP, Hôpital Lariboisière, Physiologie Clinique – Explorations Fonctionnelles, Hypertension Unit, F-75010 Paris, France; 2 Université de Paris Cité, INSERM UMR1148 - LVTS, Hôpital Bichat, F-75018 Paris, France

**Keywords:** Risk factors, Diseases

## Abstract

In developed countries, aortic coarctation (AC) is generally diagnosed by fetal echocardiography during the third trimester of pregnancy, or during the neonatal period based on the absence of femoral pulses or the presence of a left supraclavicular systolic murmur. However, AC may be diagnosed late, such as in adult migrants arriving from developing countries without documented medical history although they may require healthcare support during their stay. We report three cases of the incidental diagnosis of thoracic aortic malformations in adults (27, 38 and 43 years) referred for the management of uncontrolled high blood pressure, with major cerebrovascular events for the two oldest. Doppler ultrasound imaging indicated for suspected renal artery stenosis and atheroma lesions revealed abnormal lower-body and normal upper-body arterial blood flow velocity waveforms constitutive of a pathognomonic hemodynamic pattern of AC, a diagnostic which was in all three cases confirmed by multidetector computed tomography-angiography. None of these patients had undergone complete cardiovascular examination, particularly with effective peripheral pulse palpation, during the period preceding the occurrence of major cardiovascular events or at any other time after birth. Our observation suggests that a simple medical examination could have prevented diagnostic wandering and, possibly, the occurrence of severe cerebrovascular complications in two of these three patients.

Patient 1 was a 27-year-old amateur basketball player referred by a sports cardiologist for the etiological assessment of uncontrolled hypertension despite triple antihypertensive therapy (20 mg olmesartan, 20 mg lercanidipine, and 12.5 mg hydrochlorothiazide). The patient presented weakness of the lower limbs, NYHA II exertional dyspnea, and atypical chest pain. Doppler ultrasound imaging of the carotid and subclavian arteries displayed normal blood flow velocity waveforms. The walls of the abdominal aorta and renal, digestive, and lower-limb arteries were normal, whereas the abdominal infra renal aorta was thin, with an external diameter of 13 mm. Pulsed Doppler ultrasound revealed “*tardus-parvus*” patterns of the blood flow velocity waveforms in the abdominal aorta and all side and terminal branches. A “*tardus-parvus*” pattern is a modification in the spectral analysis of the blood flow velocity waveform that combines three criteria: an enhanced systolic rise-time (by >0.07 s), a decrease in peak systolic velocity, and a low resistive index. The internal thoracic arteries, side branches of the subclavian arteries, and the epigastric arteries were dilated and anastomosed at epigastria. Pulsed Doppler ultrasound showed high levels of anterograde blood flow in the internal thoracic arteries and high levels of retrograde blood flow in the epigastric arteries supplying the femoral arteries (Fig. 1A–G). The ankle brachial index was low, at 95/145 mmHg. Electrocardiogram-gated multidetector computed tomography angiography, confirmed AC with tight stenosis at the aortic isthmus and the development of numerous large collateral arteries around the stenosis (Fig. 1H). The patient underwent very late endovascular treatment (60 weeks after diagnosis), consisting of the implantation of an uncovered stent in the thoracic aorta, with a good anatomical and functional outcome. This therapeutic endovascular procedure was preferred over end-to-end aortic anastomosis because of the presence of numerous large collateral vessels bypassing at the site of the AC [1]. This treatment restored normal blood flow velocity waveforms patterns in all abdominal and lower-limb arteries, reflecting the restoration of normal blood pressure perfusion regimes. The antihypertensive treatment was then eased, and blood pressure was controlled with antihypertensive bi-therapy (olmesartan and hydrochlorothiazide). The long time period between diagnosis and endovascular treatment was due a one-year stay of the patient in his country of origin.

**Fig. 1:**
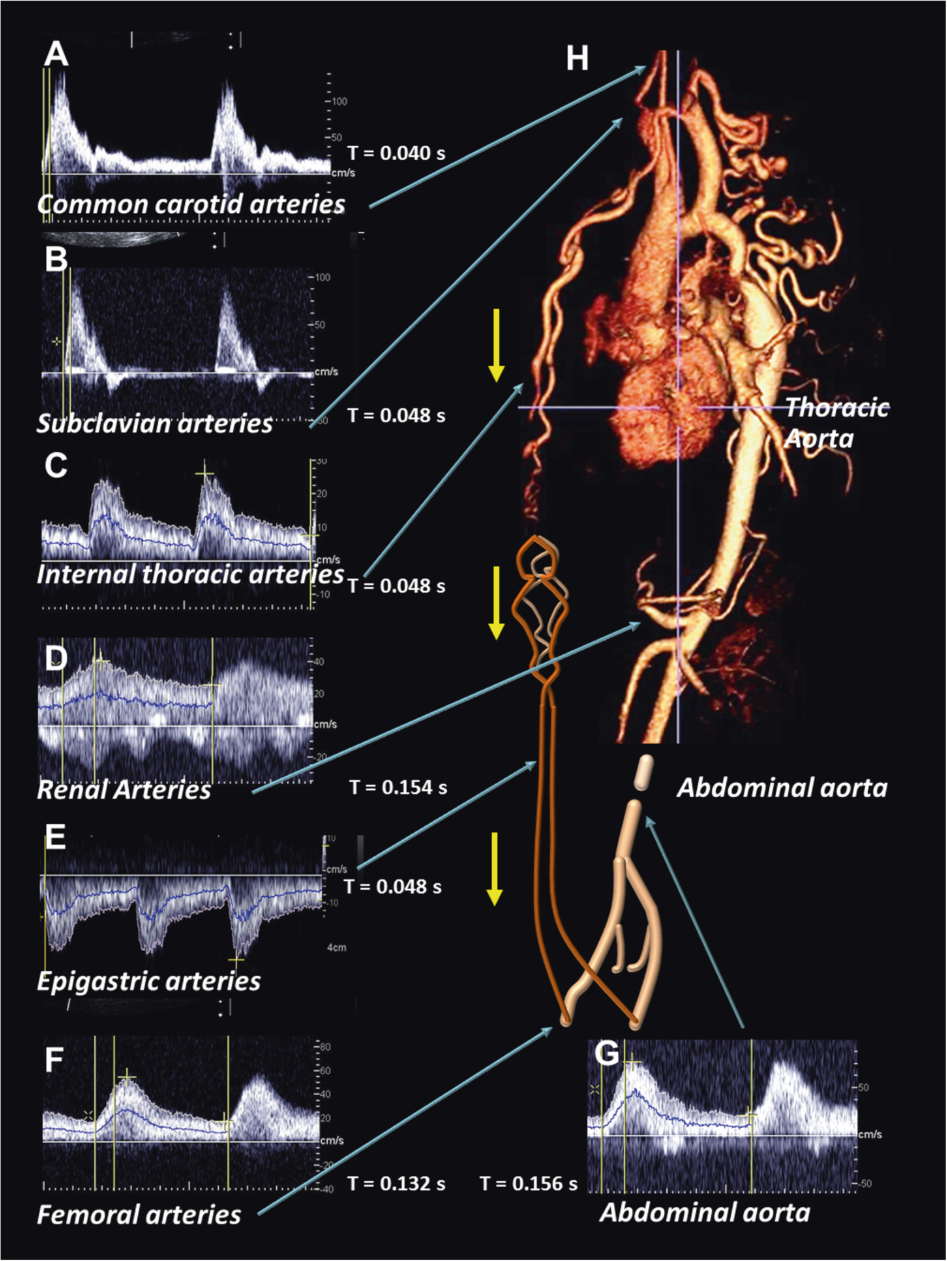
Doppler ultrasound imaging. Blood flow velocity waveforms recorded, **A** for the common carotid artery, (**B**) the subclavian artery, (**C**) the internal thoracic artery, (**D**) the renal artery, (**E**) the epigastric artery, (**F**) the common femoral artery and (**G**) the abdominal aorta, (**H**) top, multidetector computed tomography angiography with three-dimensional reconstructed images before endovascular treatment; bottom, schematic representation of the end of the aorta, the epigastric artery, the iliac and femoral arteries. Blood flow velocity waveforms were normal in the common carotid and subclavian arteries, but displayed a “*tardus-parvus*” pattern in the abdominal aorta, as in the renal and common femoral arteries. Blood flow velocities were high in the internal thoracic and epigastric arteries, epigastric arteries presented reverted flows towards the common femoral arteries. Yellow arrows indicate the direction of blood flows in the internal thoracic and epigastric arteries.

Patient 2 was 38-year-old with uncontrolled hypertension hospitalized for an inaugural ischemic stroke. The neurology department subsequently referred this patient for Doppler ultrasound imaging to check for renal artery stenosis and for carotid atheroma. The patient’s medical history included hypertension with mild hypokalemia (3.3 mmol.L^−1^) treated with dual therapy (20 mg lisinopril and 12.5 mg hydrochlorothiazide), heterozygosity for the sickle cell trait, and the use of nasal vasoconstrictors one month before the stroke. Doppler ultrasound imaging of carotid, subclavian, internal thoracic, abdominal aortic, renal and femoral arteries revealed similar hemodynamic patterns as in patient 1. A diagnosis of AC was, therefore, suspected and confirmed by multidetector computed tomography angiography. Unfortunately, the patient suffered a lethal hemorrhagic transformation of his ischemic stroke.

Patient 3 was 43-year-old referred by the neurology department for etiological assessment of severe, poorly balanced arterial hypertension, treated with 50 mg nicardipine twice daily, complicated by a vertebrobasilar stroke and cardiac arrest, from which the patient recovered. Doppler ultrasound imaging revealed similar hemodynamic patterns as in patients 1 & 2. The ankle brachial index was low, at 105/173 mmHg. Multidetector computed tomography angiography showed a type A interrupted aortic arch associated with dilation of the supra-aortic trunks and all intercostal arteries. The patient underwent late (96 weeks after diagnosis) but successful end-to-end aortic anastomosis. This surgical procedure restored normal blood flow velocity waveforms in all the abdominal and lower-limb arteries, but arterial hypertension requiring pharmacological treatment (1 mg rilmenidine, 16 mg candesartan, 10 mg amlodipine, 5 mg nebivolol) persisted. The long time interval between diagnosis and surgical treatment was due to the administrative difficulties often faced by migrant workers seeking medical care in France.

The hemodynamic presentation common to all three patients on ultrasound examination was the presence of a “*tardus-parvus*” pattern of all blood flow velocity waveforms for all abdominal visceral and lower-limb arteries associated with normal blood flow velocity waveforms for the cephalic and upper-limb arteries. The “*tardus-parvus*” pattern due to the presence of an upstream tight arterial stenosis consists of enhanced systolic rise-time (by >0.07 s), a decrease in peak systolic velocity, and low resistive indices related to downstream arteriolar vasodilation as an adaptation to the decrease in hemodynamic pressures. The existence of a “*tardus-parvus*” pattern in both renal arteries was previously reported to be due to the presence of an upstream AC in two boys (11- and 15 years of age) [2] and in one 19-year-old man [3], but such a pattern has to our knowledge not been reported for adult patients at the time of a major cardiovascular event. The combined results of Doppler ultrasound imaging on the abdominal and lower-limb arteries (below the diaphragm) and on the cephalic and upper-limb arteries (above the diaphragm) led to the suspicion of aortic stenosis, even though the origin of the patients’ clinical signs had remained unidentified since birth. Definitive diagnosis was obtained by multidetector computed tomography angiography or magnetic resonance angiography of the thorax and abdomen. AC or interrupted aortic arch is rarely diagnosed in adults in developed countries, as perinatal screening effectively detects such aortic abnormalities. Type A interrupted aortic arch may be the ultimate stage of AC, given the reports of cases of AC progressing to interrupted aortic arch [4]. Its causes are poorly understood and are probably multifactorial and interrelated. According to the Skodaic hypothesis, they may include true malformations due to embryogenic abnormalities and the presence of aberrant ductal tissue in the aortic wall at the aortic isthmus, leading to isthmic stenosis on retraction at birth [5]. Certain familial forms of AC and the association of AC with several syndromic conditions, such as Turner syndrome, suggest a possible genetic component. The *NOTCH1* gene, encoding a protein involved in cardiac development and vasculogenesis, may also be involved [6]. Less than a third of cases of AC are diagnosed on check-up ultrasound scans during pregnancy [7]. This highlights the importance of clinical examination of the newborn, including the verification of the femoral pulses during the first three days after birth.

Aortic coarctation correction may not normalize blood pressure as the mechanisms driving hypertension are partially independent. Endothelial dysfunction is systematic and may underlie the persistence of hypertension even after surgical or endovascular treatment [8]. Furthermore, changes in oscillatory shear indices and wall shear stress, particularly in the coronary and carotid arteries, entail a risk of atherosclerosis, leading to major cardiovascular and cerebrovascular events in the absence of early diagnosis and correction of the aortic malformation. In this case, such events occurred in the two oldest of the three patients [9]. Early and accelerated arterial stiffening in these patients may also increase the risk of adverse cardiovascular and cerebrovascular events [10].

All three patients were of African origin, native to Africa. In West and Central Africa, only one in two women gives birth in healthcare facilities. Effective clinical examinations at birth are therefore lacking for most infants. Pregnant women also have limited access to antenatal gynecological consultations. We would recommend that individuals originating from developing countries that have a history of walking difficulties from childhood and present with arterial hypertension in adulthood should systematically undergo examination for unidentified aortic malformations. The first step in this process should be the verification of femoral pulses. If undetectable, Doppler ultrasound examinations should be performed and followed by multidetector computed tomography angiography or magnetic resonance-angiography. In addition, we would recommend that such patients undergo specific assessments for congenital heart diseases, such as tetralogy of Fallot [11], pulmonary stenosis [12], transposition of the great arteries [13] and other extra cardiac congenital diseases. Late diagnosis, due to the clinician’s lack of awareness of the disorder, can expose the patient to the risk of major complications and impaired long-term survival.
